# Do previous percutaneous renal procedures affect the outcome of ultrasound-guided PCNL? A study at a large-volume center

**DOI:** 10.3389/fsurg.2026.1721410

**Published:** 2026-04-07

**Authors:** Xue Zeng, Yang Chen, Sen Lin, Yangyang Xu, Zhichao Luo, Wenjie Bai, Jianxing Li, Bo Xiao

**Affiliations:** Department of Urology, Beijing Tsinghua Changgung Hospital, School of Clinical Medicine, Tsinghua Medicine, Tsinghua University, Beijing, China

**Keywords:** kidney, percutaneous nephrolithotomy, previous, stone, ultrasound

## Abstract

**Purpose:**

This study aimed to investigate the influence of previous percutaneous renal procedures on the outcome of ultrasound (US)-guided percutaneous nephrolithotomy (PCNL) in patients with upper urinary tract stones.

**Patients and methods:**

Patients with a history of percutaneous renal procedures (PCNL/renal nephrostomy) from July 2017 to June 2023 were enrolled in this study as Group 1 (*n* = 77). Patients who underwent PCNL during the same period and had no history of percutaneous renal puncture surgery were enrolled in Group 2 (*n* = 160). All the procedures were performed under US guidance. Standard access (22–24 Fr) was achieved in all patients in the prone position. Relevant patient characteristics, operative variables, and postoperative data were collected and analyzed, focusing on the stone-free rate (SFR) and complications.

**Results:**

The procedure was successful in all patients. The patients’ basic characteristics (gender, age, BMI, and stone size) were similar between the two groups. Puncture time and access creation time were significantly longer in Group 1 than in Group 2 (*p* = 0.02, *p* = 0.01). Similarly, Group 1 demonstrated a significantly higher number of access tracts compared to Group 2 (*p* = 0.02). The final SFR in Group 1 showed no significant difference compared to Group 2 (*p* = 0.09). Operative duration in Group 1 was longer than in Group 2 (*p* = 0.1). Postoperative hospitalization, hemoglobin loss, transfusion rate, embolization rate, and overall complication rate were not significantly different between the two groups.

**Conclusion:**

Ultrasound-guided PCNL is safe and effective for patients with prior PCNL history, demonstrating an acceptable SFR. However, these cases exhibited prolonged access creation time, increased operative duration, and required more surgical tracts compared to naive cases.

## Introduction

The management of upper urinary tract stones has evolved substantially with the advancement of percutaneous nephrolithotomy (PCNL), which remains the gold standard treatment for large or complex renal calculi (1). Recent guidelines emphasize the growing adoption of ultrasound (US)-guided access techniques over traditional fluoroscopy, driven by its radiation-free nature and real-time visualization of critical structures (2). However, a distinct clinical challenge arises when addressing patients with a history of percutaneous renal interventions, including prior PCNL or nephrostomy placement. Anatomical distortions from fibrosis, scar formation, or an altered pelvicalyceal architecture in these “redo” cases may theoretically compromise procedural efficacy and safety. However, evidence to guide practice remains sparse (3).

Abnormal anatomical factors serve as both underlying contributors to stone formation and significant determinants of stone recurrence. In China, open nephrolithotomy has been largely replaced by PCNL over the past two decades. However, regional disparities in PCNL implementation, diverse stone complexity, and postoperative complications have introduced substantial intrarenal structural abnormalities in such patients. While the literature broadly supports the safety and efficacy of repeat PCNL in cases with prior intervention, inconsistencies persist regarding clinical outcomes (4, 5). Crucially, whether US-guided PCNL achieves comparable results in this high-risk population remains underexplored, necessitating dedicated investigations to validate its role in managing anatomically distorted collecting systems.

Thus, we conducted a comparative cohort study analyzing the perioperative outcomes of US-guided PCNL in patients with vs. without prior percutaneous renal procedures. This investigation aims to provide clinically actionable insights to optimize preoperative planning and to explore the safety profile and technical characteristics of secondary PCNL procedures.

## Patients and methods

### Study design and population

A total of 237 patients who underwent US-guided PCNL at our center were retrospectively analyzed from July 2017 to June 2023. Informed consent from the participants was obtained from the IRB of the Beijing Tsinghua Changgung Hospital ethics committee. The inclusion criteria were as follows: age 18–70 years, stone burden >2 cm in maximal diameter, indication for ultrasound-guided PCNL, tract size standardization (22–24 Fr), and previous history of PCNL or percutaneous nephrostomy permitted. The exclusion criteria were as follows: prior open or laparoscopic renal surgery, coagulation disorders, congenital renal anomalies (e.g., horseshoe kidney, polycystic kidney disease, or ectopic kidney), and spinal-pelvic anatomical deformities. Among the cohort, 77 patients had undergone prior percutaneous renal interventions (including PCNL and percutaneous nephrostomy), while 160 patients had no history of these procedures. The baseline demographic and clinical characteristics of the study population were recorded. Intraoperative parameters and postoperative outcomes were systematically documented. 17G puncture needle (Urotech, Germany) was employed for the puncture. Upon successful access, a J-tipped guidewire was advanced into the target calyx, with its position confirmed in real time via ultrasound. Subsequently, a 24Fr or 22Fr tract was established using an Alken coaxial metal dilator (Richard Wolf, GmbH). After tract establishment, an 18Fr nephroscope was used in all cases to verify tract positioning. At the conclusion of the procedure, a 14Fr nephrostomy tube was placed in each tract, and a 6Fr double-J stent was indwelled for 2–4 weeks. The nephrostomy tube was removed 3–5 days postoperatively. All procedures were performed by a single surgeon with extensive experience (primary operator in >3,000 US-guided PCNL cases).

The size of the calculi was determined by measuring their maximum diameter. The primary endpoint was the stone-free rate (SFR) in 1 month. Stone-free data was classified into three grades: Grade A (no stones on CT scan), absolute stone-free, Grade B (Grade A stones plus ≤2 mm fragments) relative stone-free, and Grade C (Grade A and B stones plus C 2.1 – 4 mm fragments) fragments relative stone-free. Secondary outcome measures included operative time (from the completion of access tract creation to the placement of a nephrostomy tube), percutaneous puncture time, access creation time, postoperative blood loss (assessed by perioperative hemoglobin decline), and postoperative complication rates (assessed using the Clavien–Dindo classification system). Renal function (serum creatinine), stone composition, and other related parameters were also recorded.

### Statistical analysis

Continuous data are presented as the mean ± standard deviation (m ± SD), and between-group data were compared using a one-way analysis of variance. Categorical data are presented as numbers and/or percentages and were analyzed using the χ^2^ test or Fisher's exact test. All statistical analyses were performed using SPSS version 20.0 (IBM Corp., Armonk, NY, USA). A *p*-value <0.05 was considered statistically significant.

## Results

A total of 237 patients underwent ultrasound-guided PCNL, with 77 in Group 1 (with a history of percutaneous renal interventions) and 160 in Group 2 (without a history of percutaneous procedures). In Group 1, the stones were residual stones that had not been previously targeted. The targeted calyces during the previous PCNL were different. Baseline demographics, including age (*p* = 0.98), sex ratio (*p* = 0.21), BMI (*p* = 0.73), stone size (*p* = 0.18), and surgical side, were comparable between the groups (Table 1). Notably, preoperative urine culture positivity was significantly higher in Group 1 (67.5% vs. 52.5%, *p* = 0.03). Group 1 required significantly longer percutaneous puncture time (179.1 ± 68.2 vs. 119.3 ± 78.5 s, *p* = 0.02) and access tract creation time (5.2 ± 3.5 vs. 3.6 ± 2.8 min, *p* = 0.03). Operative time was notably longer in Group 1 (81.6 ± 21.3 vs. 71.1 ± 15.3 min, *p* = 0.10), though not statistically significant. Group 1 required significantly more access tracts than Group 2 (2.3 ± 1.2 vs. 1.4 ± 0.5, *p* = 0.03). At the 1-month follow-up, no significant disparity was observed in SFR between the groups (Grade A: 23.4% vs. 26.3%, Grade B: 41.6% vs. 50.6%, Grade C: 62.3% vs. 76.3%; overall *p* = 0.07). The overall SFR (absolute stone-free + residual fragments ≤4 mm) in Group 1 was 62.3%, while the SFR in Group 2 was 76.3%. Renal function, as assessed by pre- and postoperative serum creatinine levels, remained stable and comparable between the groups (*p* = 0.76 and *p* = 0.68, respectively). Hemoglobin decline (15.7 ± 8.4 vs. 13.6 ± 7.7 g/L, *p* = 0.38) and transfusion rates (6.5% vs. 5%, *p* = 0.21) did not differ significantly. Postoperative hospitalization durations were similar (5.3 ± 4.6 vs. 5.7 ± 2.6 days, *p* = 0.29). Complications classified using the Clavien–Dindo criteria revealed no significant disparity in overall rates (Clavien 1: 19.5% vs. 20.0%; Clavien 2: 22.0% vs. 19.4%; *p* = 0.72). Calcium-containing stones predominated in both groups (55.8% vs. 61.3%), followed by non-calcium calculi (44.2% vs. 38.7%), with no significant difference in composition (*p* = 0.42). No patient underwent any ancillary procedures or re-intervention within 3 months after their operation (Tables 2, 3).

**Table 1:** Cohort patient characteristics.

Variable	Group 1 (*n* = 77)	Group 2 (*n* = 160)	*P*-value
Age (year), mean (SD)	49.1 (16.2)	47.9 (13.9)	0.98
Sex ratio (F/M)	39/38	88/72	0.21
BMI (kg/m^2^), mean (SD)	27.1 (8.8)	24.9 (7.6)	0.73
Surgical side (Rt/Lt)	39/38	86/74	0.19
Stone size (cm), mean (SD)	3.9 (2.6)	4.4 (2.9)	0.18
Previous renal surgery, *n* (%)			
PCNL	50 (64.9)	–	–
Percutaneous nephrostomy	27 (35.1)		
Urine culture, *n* (%)			
Positive	52 (67.5)	84 (52.5)	0.03
Negative	25 (32.5)	76 (47.5)	
Guy's stone score *n* (%)			
I	5 (6.5)	10 (6.25)	0.65
II	29 (37.7)	51 (31.9)	
III	34 (44.2)	71 (44.4)	
IV	9 (11.9)	28 (17.5)	

**Table 2:** Intraoperative parameters.

Variables	Group 1 (*n* = 77)	Group 2 (*n* = 160)	*P*-value
Puncture time (s), mean (SD)	179.1 (68.2)	119.3 (78.5)	0.02
Tract creation time (min), mean (SD)	5.2 (3.5)	3.6 (2.8)	0.03
Operative time (min), mean (SD)	81.6 (21.3)	71.1 (15.3)	0.10
Number of access tracts, mean (SD)	2.3 (1.2)	1.4 (0.5)	0.03

**Table 3:** Postoperative outcomes.

Variables	Group 1 (*n* = 77)	Group 2 (*n* = 160)	*P*-value
SFR, *n* (%)			
Grade A	18 (23.4)	42 (26.3)	0.07
Grade B	32 (41.6)	81 (50.6)	
Grade C	48 (62.3)	122 (76.3)	
Mean decline in Hb level, g/L, mean ± SD	15.7 (8.4)	13.6 (7.7)	0.38
Preoperative serum creatinine level, mg/dL, mean (SD)	1.4 (0.5)	1.3 (0.4)	0.76
Postoperative serum creatinine level, mg/dL, mean (SD)	1.6 (0.6)	1.4 (0.6)	0.68
Postoperative hospitalization time (d), mean (SD)	5.3 (4.6)	5.7 (2.6)	0.29
Complications, *n* (%)			
Clavien 1	15 (19.5)	32 (20.0)	0.72
Fever	15 (19.5)	32 (20.0)	
Clavien 2	17 (22.0)	31 (19.4)	
Antibiotics	17 (22.0)	31 (19.4)	
Sepsis	7 (9.1)	9 (5.6)	
Blood transfusion	5 (6.5)	8 (5)	
Stone composition, *n* (%)			
Calcium-containing	43 (55.8)	91 (61.3)	0.48
Non-calcium-containing	34 (44.2)	69 (38.7)	

## Discussion

Previous renal surgeries, whether open or percutaneous, frequently result in significant structural disruption and anatomical remodeling of the kidney and its surrounding tissues. Historically, open renal procedures are known for extensive destruction of the renal and perirenal architecture, leading to pronounced perinephric scarring, marked fibrosis, and distorted pelvicalyceal anatomy (6, 7). These interventions often precipitate infundibular stenosis and calyceal displacement, giving rise to three primary clinical challenges.

First, fibrotic resistance increases operative complexity. The presence of retroperitoneal, perirenal, and muscular scar tissue creates substantial resistance to needle advancement, which can impede trajectory control and compromise targeting precision during renal puncture. Furthermore, the technical difficulty of navigating these fibrotic layers often prolongs tract establishment and extends overall operative duration (8). Second, structural alterations can reduce clearance efficacy. Distortions in the collecting system—particularly infundibular stenosis—increase intraoperative resistance during nephroscope manipulation. This limitation not only restricts the efficacy of stone clearance via a single tract but also elevates the risk of infundibular tears, collectively contributing to suboptimal SFRs (9, 10). Third, vascular remodeling heightens bleeding risk. Postoperative changes in the renal vasculature can induce anomalous arterial distributions and regional perfusion imbalances. These factors increase the likelihood of vascular injury during tract dilation and the potential for severe postoperative hemorrhage (11). Ultimately, the combination of renal scarring and chronic inflammatory changes significantly exacerbates the risk of perioperative bleeding.

In recent decades, advancements in PCNL have shifted clinical practice toward minimally invasive techniques, with the majority of patients now undergoing PCNL or nephrostomy rather than open surgery (12). Although PCNL is widely regarded as less destructive to renal and perirenal structures compared to open approaches, our institutional experience highlights critical challenges. Due to significant variations in surgical skills among physicians across different regions and as China's designated training and referral center for urinary tract stone management, we frequently encounter patients in clinical practice who develop severe complications following PCNL procedures performed at external hospitals. Many of these patients had undergone one or several unsuccessful “minimally invasive” procedures, frequently complicated by intraoperative hemorrhage requiring termination, postoperative subcapsular hematomas, residual stone burdens, and persistent febrile morbidity. Notably, PCNL itself may induce adverse structural changes, including fluid extravasation-induced fibrosis, access tract scarring, and cicatrization of the collecting system, all of which may impede subsequent interventions. Furthermore, the cumulative impact of these alterations on renal functional reserve remains poorly quantified.

Previous studies investigating the impact of prior renal surgery on PCNL outcomes have yielded inconsistent results. Margel et al. compared 21 patients who underwent PCNL following open nephrolithotomy on the same kidney with a control group; they found that while these cases required longer operative times and a higher rate of auxiliary procedures, morbidity and efficacy remained comparable (13). Conversely, Gulani et al. demonstrated that PCNL is a safe and effective treatment modality regardless of a history of prior PCNL or open renal surgery (14). In contrast, Güzel et al. reported that while PCNL is generally safe, a history of open renal surgery—though not prior PCNL—significantly reduced the success rate of subsequent procedures (15). Notably, the aforementioned studies exclusively utilized fluoroscopic (X-ray) guidance; to date, there is no published evidence confirming whether ultrasound (US)-guided PCNL yields similar outcomes. Unlike X-ray guidance, US-guided PCNL relies heavily on real-time imaging of perirenal tissues to ensure puncture accuracy. Perirenal scarring or fibrotic changes may deflect the needle trajectory or compromise ultrasonic visibility, thereby increasing procedural complexity. To our knowledge, this study is the first to evaluate the efficacy and safety of total US-guided PCNL specifically in patients with a history of prior percutaneous renal surgery.

In this study, we observed that the percutaneous renal surgery group exhibited a significantly higher rate of positive urine cultures compared to the non-surgical group (67.5% vs. 52.5%, *p* = 0.03). This disparity may be attributed to more complex stone burdens and the presence of indwelling drainage tubes or stents in the surgical cohort, as previous studies have demonstrated that ureteral stents or nephrostomy catheters increase the risk of urinary tract infections, concordant with our findings. Needle puncture time—defined as the interval from skin penetration to successful calyceal access—and tract establishment time—defined as the period from guidewire insertion to final sheath placement—were both significantly prolonged in Group 1 (179.1 ± 68.2 vs. 119.3 ± 78.5 s; 5.2 ± 3.5 vs. 3.6 ± 2.8 min). Renal surgery leads to retroperitoneal scarring around the kidney that may adversely affect the introduction of the access needle and prevent proper dilatation of the tract in subsequent PCNL procedures. Notably, the routine use of sequential fascial dilators for tract creation revealed substantial resistance during dilation. Under ultrasound guidance, the inability to monitor real-time dilation depth exacerbated the difficulty in achieving precise tract alignment. In some cases, multiple dilation attempts were required to establish an optimal working tract, further contributing to prolonged tract creation times. However, the mean operative time was longer in patients with previous percutaneous procedures, but this was not statistically significant. Regarding the number of operative tracts, Group 1 required a significantly greater number compared to Group 2 (2.3 ± 1.2 vs. 1.4 ± 0.5, *p* = 0.03), likely attributable to their prior PCNL procedures. There were no statistically significant differences observed between the two groups in postoperative hemoglobin decline, transfusion rate, renal function parameters, or overall complication rate. We hypothesize these comparable outcomes may reflect the surgeon's expertise in maintaining low complication rates despite significant inter-group differences in the number of access tracts and tract creation time.

The process of scarring after renal surgery may also occur within the collecting system and may affect the rate of kidney stone clearance. In many patients who undergo PCNL, previous interventions often focused on removing easily accessible stones while leaving residual calculi in less accessible calyces. This selective stone removal, coupled with the inevitable disruption of the collecting system architecture, significantly elevates the technical challenges of subsequent procedures for residual calculus management. In our study, Group 1 exhibited a lower SFR than Group 2 (62.3% vs. 76.3%), but the difference was not statistically significant (*p* = 0.07). It is important to note that several parameters in our study yielded *p*-values that approached, but did not reach, the conventional threshold of *p* < 0.05. These results must be interpreted with caution. The lack of statistical significance in these instances may stem from the study being underpowered to detect subtle or moderate clinical differences (Type II error), rather than a true lack of effect. Consequently, these findings should be considered exploratory, and larger prospective trials are required to provide more definitive evidence. Some studies suggest that balloon dilation is advantageous for patients with retroperitoneal scarring (16). However, we contend that in non-hydronephrotic kidneys, balloon-assisted tract establishment introduces unpredictability. In this cohort, sequential metal dilators combined with a two-step approach were employed to standardize tract creation, achieving favorable outcomes.

This study had several limitations. First, as a retrospective analysis, it is inherently susceptible to selection bias and lacks randomization, particularly due to the relatively small cohort of patients who underwent PCNL during the early stage of this research, which may have compromised the reliability of the findings. The second limitation is the heterogeneity within Group 1, which included patients who had undergone prior PCNL and those with a pre-existing nephrostomy. Prior renal surgery can lead to perirenal fibrosis and distorted pelvicalyceal anatomy, potentially increasing the technical difficulty of secondary access. Conversely, a pre-existing nephrostomy may provide better preoperative drainage but could also be a surrogate for more complex or recurrent stone disease. While our subgroup analysis did not reveal a statistically significant impact, this potential bias should be considered when interpreting our results. Third, the exclusion of patients with a history of open renal surgery represents a critical gap, as this population often exhibits significant renal anatomical variations and tissue scarring/fibrosis. Fourth, this study only included 237 patients, and it must be acknowledged that all the surgical procedures in this study were performed by a single, highly experienced surgeon with proven expertise in managing complex renal calculi at a high-volume tertiary center. While this design minimized technical confounding variables and ensured consistency across the study cohorts, it may limit the generalizability of our results. The outcomes observed here may not be directly reproducible in clinical settings with surgeons of varying experience levels or during the early stages of the learning curve for PCNL. Future multicenter studies involving multiple operators with different levels of expertise and a greater number of included patients are warranted to further validate the broader applicability of our findings. Whether these factors adversely impact PCNL outcomes remains to be investigated in future studies.

## Conclusion

US-guided PCNL achieves acceptable efficacy and safety in patients with prior percutaneous renal interventions, despite the challenges posed by fibrotic anatomy. While longer access times and additional tracts reflect procedural complexity, the absence of a significant difference in SFR or complication rate underscores the adaptability of US-guided strategies. Future research should integrate long-term functional outcomes and standardized protocols for managing anatomically hostile systems.

## Data Availability

The original contributions presented in the study are included in the article/Supplementary Material, further inquiries can be directed to the corresponding author.
